# Scaling multi-instance support vector machine to breast cancer detection on the BreaKHis dataset

**DOI:** 10.1093/bioinformatics/btac267

**Published:** 2022-06-27

**Authors:** Hoon Seo, Lodewijk Brand, Lucia Saldana Barco, Hua Wang

**Affiliations:** Department of Computer Science, Colorado School of Mines, Golden, CO 80401, USA; Department of Computer Science, Colorado School of Mines, Golden, CO 80401, USA; Department of Computer Science, Colorado School of Mines, Golden, CO 80401, USA; Department of Computer Science, Colorado School of Mines, Golden, CO 80401, USA

## Abstract

**Motivation:**

Breast cancer is a type of cancer that develops in breast tissues, and, after skin cancer, it is the most commonly diagnosed cancer in women in the United States. Given that an early diagnosis is imperative to prevent breast cancer progression, many machine learning models have been developed in recent years to automate the histopathological classification of the different types of carcinomas. However, many of them are not scalable to large-scale datasets.

**Results:**

In this study, we propose the novel Primal-Dual Multi-Instance Support Vector Machine to determine which tissue segments in an image exhibit an indication of an abnormality. We derive an efficient optimization algorithm for the proposed objective by bypassing the quadratic programming and least-squares problems, which are commonly employed to optimize Support Vector Machine models. The proposed method is computationally efficient, thereby it is scalable to large-scale datasets. We applied our method to the public BreaKHis dataset and achieved promising prediction performance and scalability for histopathological classification.

**Availability and implementation:**

Software is publicly available at: https://1drv.ms/u/s!AiFpD21bgf2wgRLbQq08ixD0SgRD?e=OpqEmY.

**Supplementary information:**

Supplementary data are available at *Bioinformatics* online.

## 1 Introduction

Every year, approximately 250 000 women in the United States are diagnosed with breast cancer (CDC, 2020). Differentiating between the different types of carcinomas (ductal, lobular, mucinous and papillary) is essential for making an accurate diagnosis. Histopathology allows for this close examination that leads to patients receiving a personalized treatment and can increase their likelihood of survival. Histopathology is the examination of tissue sections with a microscope to aid in the diagnosis of illnesses such as cancer and inflammatory diseases and increase the likelihood of survival. These tissue sections can also be called whole-slide images (WSIs) or histopathological images when they are digitized. Traditionally, clinical disciplines such as radiology and pathology have relied heavily on specialized training to detect the presence of these diseases in histopathological images. A diagnosis is based on features exhibited by tissue samples on a cellular level. An anomaly in the cell architecture and the presence or absence of certain biological attributes can be strong indicators of a particular disease. For example, abnormal cells that divide uncontrollably, also known as carcinomas, lead to a cancer diagnosis when detected. A pathologist can detect this abnormal growth/tumor from a histopathological image and assess which regimen should be prescribed to halt the progression of the disease. This pattern analysis is an essential component of precision medicine, since it makes a diagnosis based on patient-specific histopathological images.

Modern medical procedures and technologies have increased the number of biopsies performed, and consequently, the number of histopathological images collected has increased far beyond the reasonable workload of pathologists (van der Laak *et al.*, 2021). This poses an impediment to precision medicine, since it requires the analysis of vast amounts of medical data. However, recent advancements in the field of artificial intelligence have shown promise in automating the analysis of histopathological images and improving the accuracy and speed of a diagnosis. Just as a pathologist finds patterns that help detect cellular abnormalities, algorithms can be used to extract features from an image such as pixel intensity (Hamilton *et al.*, 2007), texture (Haralick, 1979) and Zernike moments (Khotanzad and Hong, 1990). The application of computational algorithms to diagnostic fields can aid pathologists in drawing accurate and precise conclusions in an efficient and reproducible manner (Gurcan *et al.*, 2009).

**Fig. 1.**
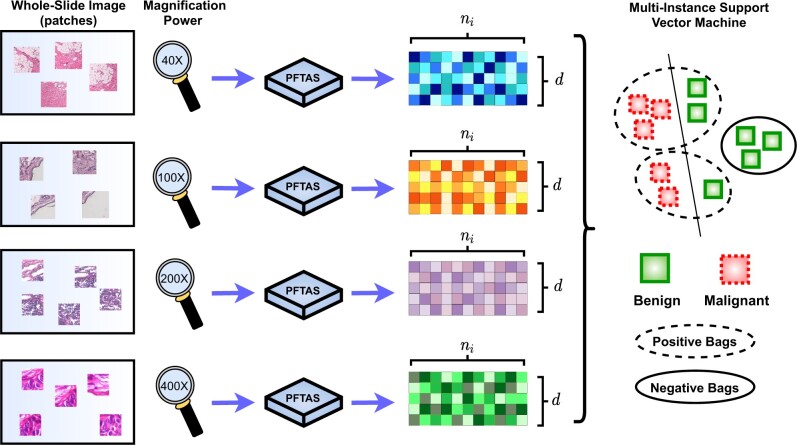
A visualization of our processing pipeline for our MIL algorithm applied to the BreaKHis dataset. We sample the patches (instances) from the histopathological images (bags) of four different magnification levels, and process the patches with PFTAS method which results in *d*-features vectors of *n_i_* instances for each image. Finally, our multi-instance SVM classify the bags as malignant or benign

In our research, we focused our analysis efforts on developing a classification model for the public BreaKHis dataset (Spanhol *et al.*, 2015), which is composed of 7909 histopathological images of different types of benign and malignant breast cancer tumors. This dataset has been instrumental in our work, since its structure allows for extensive and precise classification of histopathological images. The dataset is split into benign and malignant categories, and these are further subdivided into different types of carcinomas. The WSIs in each tumor type group are then amplified to four different magnification factors, and they are usually segmented into the patches because of their large size. As a result, the classification problem is naturally formulated as a multi-instance learning (MIL) problem (Brand *et al.*, 2021a,b; Wang *et al.*, 2011) to determine which segments of tissue in an image exhibit an indication of an abnormality. MIL is an area of machine learning in which training and testing data are organized into sets of instances known as bags. MIL is a weakly supervised learning algorithm, which means that the data are frequently provided at the bag level instead of the instance level, therefore clinicians do not need to spend a lot of resources into characterizing each image in the training dataset obtained from a biopsy. Doctors only need to label/diagnose the bag or patient as malignant and benign, and the rest of the instances or histopathological images follow suit. Despite being a very powerful approach, MIL remains a challenging problem as many standard machine learning approaches rely on fixed-length vector input which are not applicable to the dataset with a varying number of instances per bag. At the same time, MIL models should be translation invariant against the instances of each set input; the prediction of model should not be affected by the order of instances. In our work, breast cancer histopathological images are represented by a bag (set) of patches, as illustrated in Figure 1. The bags, or images, are labeled as either malignant or benign while the instances, or patches, remain unlabeled (Brand *et al.*, 2021a,b; Wang *et al.*, 2011). Taking these facts into account, we propose the Primal-Dual Multi-Instance SVM (*pdMISVM*) method (Brand *et al.*, 2021a), which improves the efficiency of optimization compared to the previously mentioned MIL approaches.

### 1.1 Related works

To ease the heavy workloads of pathologists, Computer Aided Diagnostics (CAD) has emerged to determine whether an image shows any indication of carcinoma and, if so, where the abnormality is located within the histopathological image. One of the widely used approaches is to use Convolutional Neural Networks (CNNs) trained by the patches extracted from WSIs. CNN is the combination of convolutional layers and consecutive fully connected layers and their concept comes from the working principle of receptive fields and neurons of the human eye and brain. Krizhevsky *et al.* (2012) has shown that the deep structure of the CNN can achieve state-of-the-art performance in image recognition tasks. Their model, AlexNet, has been successfully applied to BreakHis by Titoriya and Sachdeva (2019). However, CAD based on the CNNs still faces obstacles, because training a CNN requires a large amount of training data with the big computational burdens. These requirements make it difficult for predictive models to be combined with the CAD systems. The SVM applications as a practical alternative to deep learning models has also been studied (Zheng *et al.*, 2014). For example, SVM with sparsity inducing regularization (Kahya *et al.*, 2017) can achieve the promising accuracy higher than 90% in image classification. Although SVM models have fewer trainable parameters than deep learning models, and therefore require less time and computational cost to train, their training time and computational complexity increases rapidly as the number of input features increases (Kumar and Rath, 2015; Peng *et al.*, 2016). Another problem of the traditional SVM models is that they are single-instance learning (SIL) models, i.e. they are not able to handle the varying number of input instances, while the WSIs are usually segmented into the multiple patches (instances) because of the large size of WSIs.

In light of the above issue, multiple instance learning would be the better choice for the disease detection applications, and these types of algorithms have also been evaluated on the BreaKHis dataset previously. This is because, in a SIL model, classification becomes difficult when a single patch of insignificant region on the image is given. Meanwhile, MIL models enable the correct classification from some important patches, even if most patches do not include indication of carcinoma. To deal with the multi-instance dataset, several MIL methods have achieved satisfactory results in the past when performing similar tasks especially on the BreaKHis dataset (Sudharshan *et al.*, 2019). For example, Multiple Instance Learning Convolutional Neural Networks (MILCNN) (Sudharshan *et al.*, 2019) take a deep learning approach while others opt for an SVM-based multiple instance learning alternative. Some examples are Multi-Instance Support Vector Machine (MISVM) (Andrews *et al.*, 2002), sparse Multi-Instance Learning (sMIL) and sparse balanced MIL (sbMIL) (Bunescu and Mooney, 2007), and Normalized Set Kernel (NSK) and Statistics Kernel (STK) (Gärtner *et al.*, 2002). These are all methods that have been deemed successful at correctly labeling the bags in the testing dataset as either malignant or benign. However, despite the promising performance of MIL models, we point out that there is a lack of discussions on the scalability of MIL models or they do not scale to the large dataset. In addition, it is difficult to efficiently learn the hypothesis space of MIL models which involves with the multiple instances (Wei *et al.*, 2014). Unlike the previous existing algorithms, our approach scales well to larger datasets, which adds to its value for practical use.

### 1.2 The paper organization

In the remainder of this manuscript, we present an objective and associated solution of the novel *pdMISVM* that extends to large-scale data. We derive the optimization algorithm based on the multi-block alternating direction method of multipliers (ADMM) (Hong and Luo, 2017) to bypass the quadratic programming problem that comes from the typical SVM and MISVM models. We further improve the ADMM derivation to decrease the complexity with respect to the large number of features. Lastly, we provide an application of the proposed method to classify the bag of patches and identify disease relevant patches, which can reduce the workload of pathologists.

## 2 Materials and data sources

We perform classifications on the publicly available BreaKHis dataset (https://web.inf.ufpr.br/vri/databases/breast-cancer-histopathological-database-breakhis/). The BreaKHis dataset was built in collaboration with the P&D Laboratory in Parana, Brazil. BreaKHis was first introduced in ‘A Dataset for Breast Cancer Histopathological Image Classification’ by Spanhol *et al.* (2015). The dataset comprises 7909 microscopic biopsy images of breast tumor tissue images collected in a clinical study from January 2014 to December 2014. The dataset contains 2480 benign and 5429 malignant tissue samples. The images were collected using different magnifying factors (40×, 100×, 200× and 400×), and they were organized into these categories in the dataset. The samples were acquired from 82 patients whose data were anonymized. Samples were generated from breast tissue biopsy slides, stained with hematoxylin and eosin (HE) and collected by surgical open biopsy (SOB). They were labeled in the P&D Laboratory, and the diagnosis of each slide was determined by experienced pathologists (Spanhol *et al.*, 2016).

We segment the histopathological images into patches. In our experiments, each patch contains a 64 × 64 section of pixels and we extracted a random number (sampled from {1, 5, 10}) of patches for each of the images. However, in the raw tissue segments, the elements of interest such as nuclei may not be clearly visible. In light of this issue, we extract the feature vector through Parameter Free Threshold Statistics (PFTAS) (Hamilton *et al.*, 2007) for each patch. Based on experimental results of previous study (Spanhol *et al.*, 2015), PFTAS outperforms the other features such as Local Binary Patterns (LBP) (Ojala *et al.*, 2002), Completed LBP (CLBP) (Guo *et al.*, 2010), Local Phase Quantization (LPQ) (Ojansivu and Heikkilä, 2008) and Grey-Level Co-occurrence Matrix (GLCM) (Haralick *et al.*, 1973) in BreaKHis dataset. PFTAS is a method that extracts texture features by counting the number of black pixels in the neighborhood of a pixel. The total count for all the pixels in a given image is stored in a nine-bin histogram (Hamilton *et al.*, 2007). The thresholding is done by Otsu’s algorithm (Otsu, 1979) and the extractor returns a 162-dimensional feature vector. The 162 features consist of 3 channels (RGB) × 9 pixels × 3 thresholding ranges concatenated with its bitwise negated version. The Otsu’s algorithm iteratively finds the optimal threshold value by maximizing the inter-class intensity variance. As a result, PFTAS features are robust against the varying mean of intensity distribution for each RGB channel across images. To control the number of features, we concatenate several patches, and the final number of features is a multiple of 162. In our experiments, 7909 bags (images) are involved, of which 5429 are malignant and 2480 are benign.

## 3 Methods

In this section, we develop an objective for the scalable *pdMISVM* algorithm designed to handle multi-instance data. Our formulation for *pdMISVM* provides an efficient solution to avoid dependency on a quadratic programming or least-squares approach.

### 3.1 Notation

In this article, we denote matrices as **M**, vectors as **m** and scalars as *m*. The *i*th row and *j*th column of **M** are $m^{i}$ and **m**_*j*_, respectively. Similarly, $m_{j}^{i}$ is the scalar value indexed by the *i*th row and *j*th column of **M**. The matrix $M_{p}$ corresponds to the *p*th column-block of **M**. Each bag $X_{i}=\{x_{i}^{1},\ldots ,x_{i}^{n_{i}}\}$ contains *n_i_* patches and its associated label of *m*th class is represented by $y_{i}^{m}\in \{-1,1\}$.

### 3.2 A primal-dual multi-instance support vector machine

**Fig. 2.**
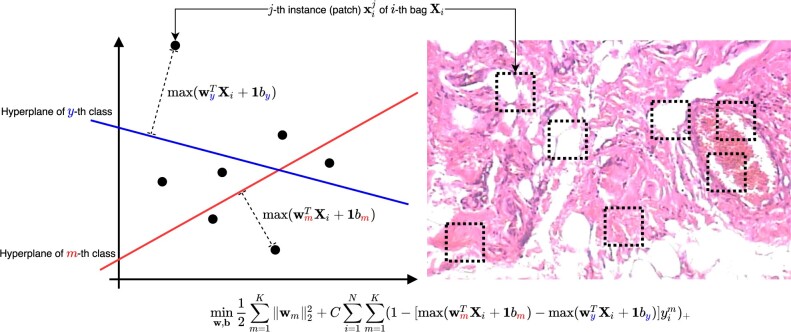
An illustration for the objective in Equation (1). In our model, each patch corresponds to each instance in a bag. We first calculate the distance from the hyperplane of each *m*th class to the farthest instance, which is the key instance triggering the bag label. By minimizing Equation (1), we optimize **W** and **b** of the hyperplanes so that the distance to the hyperplane of the correct class (*m *=* y*) is greater than the distance to the hyperplane of the incorrect (m≠y) class

The *K* class multi-instance support vector machine was proposed by Andrews *et al.* (2002), which solve the following objective:

(1) $$\underset{W,b}{\min}\frac{1}{2}\sum_{m=1}^{K}{\left|\left|w_{m}\right|\right|}_{2}^{2}+C\sum_{i=1}^{N}\sum_{m=1}^{K}(1-[\max(w_{m}^{T}X_{i}+1b_{m})-\max(w_{y}^{T}X_{i}+1b_{y})]y_{i}^{m}{)}_{+},$$

where ${\left(\cdot \right)}_{+}=\text{max}(\cdot ,0)$ and its decision function is given by:

(2) $${\tilde{y}}_{i}=\underset{m\prime }{\text{arg max}}(\max{\left(W^{T}X_{i}+b1_{i}\right)}^{m\prime }),$$

as illustrated in Figure 2.

The MISVM objective in Equation (1) is generally difficult to solve because of the coupled primal variables $w_{k}$, *b_m_* by the max(·) operations. Inspired by Nie *et al.* (2014) and Wang and Zhao (2017), we split the primal variables in Equation (1) via the ADMM approach by introducing the following constraints:

(3) $$\begin{array}{cc} \underset{W,b,E,Q,R,T,U}{\min}\; & \frac{1}{2}\sum_{m=1}^{K}{\left|\left|w_{m}\right|\right|}_{2}^{2}+C\sum_{i=1}^{N}\sum_{m=1}^{K}{\left(y_{i}^{m}e_{i}^{m}\right)}_{+}\\ \text{subject to}\; & e_{i}^{m}=y_{i}^{m}-q_{i}^{m}+r_{i}^{m},\;q_{i}^{m}=\max\left(t_{i}^{m}\right),\\ & r_{i}^{m}=\max\left(u_{i}^{m}\right),t_{i}^{m}=w_{m}^{T}X_{i}+1b_{m},\\ & u_{i}^{m}=w_{y}^{T}X_{i}+1b_{y}. \end{array}$$

From Equation (3) we derive the following augmented Lagrangian function:

(4) $$\begin{array}{cc} L_{\mu }= & \frac{1}{2}\sum_{m=1}^{K}{\left|\left|w_{m}\right|\right|}_{2}^{2}+\sum_{i=1}^{N}\sum_{m=1}^{K}C{\left(y_{i}^{m}e_{i}^{m}\right)}_{+}\\ & +\frac{\mu }{2}\sum_{i=1}^{N}\sum_{m=1}^{K}[{\left(e_{i}^{m}-(y_{i}^{m}-q_{i}^{m}+r_{i}^{m}-\lambda_{i}^{m}/\mu )\right)}^{2}\\ & +{\left(q_{i}^{m}-\max\left(t_{i}^{m}\right)+\sigma_{i}^{m}/\mu \right)}^{2}\\ & +{\left|\left|t_{i}^{m}-\left(w_{m}^{T}X_{i}+1b_{m}\right)+\theta_{i}^{m}/\mu \right|\right|}_{2}^{2}\\ & +{\left(r_{i}^{m}-\max\left(u_{i}^{m}\right)+\omega_{i}^{m}/\mu \right)}^{2}\\ & +{\left|\left|u_{i}^{m}-\left(w_{y}^{T}X_{i}+1b_{y}\right)+\xi_{i}^{m}/\mu \right|\right|}_{2}^{2}], \end{array}$$

where W,b,E,Q,T,R,U are the primal variables, Λ,Σ,Θ,Ω,Ξ are the dual variables, and μ>0 is a hyperparameter.

### 3.3 The solution algorithm

In this section, we derive the efficient solution algorithm to minimize the proposed objective in Equation (4). In Algorithm 1, we repeat the primal-dual updates until the gap in constraints from the augmented Lagrangian terms in Equation (4) becomes smaller than a predefined tolerance. In order not to distract reading attention and due to space limit, we only provide the derivation details for the class-hyperplane in $w_{m}$ and *b_m_* for each *m*th class in the main article, and leave the derivations for the other variables in the online Supplementary Appendix of this article.

**b update.** By differentiating Equation (7) element-wise with respect to *b_m_* and setting the result equal to zero, we have the following update:

(5) $$b_{m}=\underset{b_{m}}{\text{arg min}}\;\sum_{i=1}^{N}\left[{\left|\left|t_{i}^{m}-\left(w_{m}^{T}X_{i}+1b_{m}\right)+\theta_{i}^{m}/\mu \right|\right|}_{2}^{2}\right]+\sum_{i\prime =1}^{N\prime }\sum_{m\prime =1}^{K}\left[{\left|\left|u_{i\prime }^{m\prime }-\left(w_{m}^{T}X_{i\prime }+1b_{m}\right)+\xi_{i\prime }^{m}/\mu \right|\right|}_{2}^{2}\right],$$

where i′ indicates the column blocks that belong to the *m*th class are chosen from **X**. Taking the derivative of Equation (5) with respect to *b_m_*, setting the derivative equal to zero, and solving for *b_m_* gives:

(6) $$\begin{array}{c} b_{m}=\frac{\sum_{i=1}^{N}\left[t_{i}^{m}-w_{m}^{T}X_{i}+\theta_{i}^{m}/\mu \right]+\sum_{i\prime =1}^{N\prime }\sum_{m=1}^{K}[u_{i\prime }^{m}-w_{m}^{T}X_{i\prime }+\xi_{i\prime }^{m}/\mu ]}{N+KN\prime }, \end{array}$$

where N′ is the total number of patients belonging to the *m*th class.

**W update (without kernel).** We discard all terms in Equation (4) which do not include **W** and optimize the columns of **W** separately by solving the following *K* problems for m=1,…,K:

(7) $$w_{m}^{*}=\underset{w_{m}}{\text{arg min}}\;\frac{1}{2}{\left|\left|w_{m}\right|\right|}_{2}^{2}+\frac{\mu }{2}\sum_{i=1}^{N}[{\left|\left|t_{i}^{m}-(w_{m}^{T}X_{i}+1b_{m})+\theta_{i}^{m}/\mu \right|\right|}_{2}^{2}]+\sum_{i\prime =1}^{N\prime }\sum_{m\prime =1}^{K}[\frac{\mu }{2}{\left|\left|u_{i\prime }^{m\prime }-(w_{m}^{T}X_{i\prime }+1b_{m})+\xi_{i\prime }^{m\prime }/\mu \right|\right|}_{2}^{2}],$$

where N′ is the number of bags which belongs to *m*th class, and i′ denotes the indices of column blocks of **X** and the corresponding columns of **U** and Ξ. Finally $t_{i}^{m},\;\theta_{i}^{m},\;u_{i\prime }^{m\prime }$ and $\xi_{i\prime }^{m\prime }$ are row vectors corresponding to the *i*th bag and *m*th class in **T**, Θ, **U** and Ξ. By letting the derivative of Equation (7) with respect to $w_{m}$ equal zero, we attain the following closed form solution:

(8) $$\begin{array}{cc} {\left(w_{m}^{*}\right)}^{T}= & (\sum_{i=1}^{N}\left[\left(t_{i}^{m}-1b_{m}+\theta_{i}^{m}/\mu \right)X_{i}^{T}\right]\\ & +\sum_{i\prime =1}^{N\prime }\sum_{m\prime =1}^{K}[(u_{i\prime }^{m\prime }-1b_{m}+\xi_{i\prime }^{m\prime }/\mu )X_{i\prime }^{T}])\\ & *{\left(I/\mu +\sum_{i=1}^{N}X_{i}X_{i}^{T}+K\sum_{i\prime =1}^{N\prime }X_{i\prime }X_{i\prime }^{T}\right)}^{-1}. \end{array}$$

In the calculation of Equation (8) we can avoid an inverse calculation through a least-squares solver.

**W update (with kernel).** The kernel method (Shawe-Taylor *et al.*, 2004) is widely used in classification tasks to deal with non-linearity of the data. We provide the kernel extension of our method to learn the non-linear relationship between bag and target label. For the arbitrary (possibly non-linear) kernel function ϕ, we map all the columns (instances) of $X_{i}\in R^{d\times n_{i}}$ to feature vectors $\phi (X_{i})=\Phi_{i}\in R^{d_{z}\times n_{i}}$, and Equation (7) can be rewritten into:

(9) $$w_{m}^{*}=\underset{w_{m}}{\text{arg min}}\;\frac{1}{2}{\left|\left|w_{m}\right|\right|}_{2}^{2}+\frac{\mu }{2}\sum_{i=1}^{N}[{\left|\left|t_{i}^{m}-(w_{m}^{T}\Phi_{i}+1b_{m})+\theta_{i}^{m}/\mu \right|\right|}_{2}^{2}]+\sum_{i\prime =1}^{N\prime }\sum_{m\prime =1}^{K}[\frac{\mu }{2}{\left|\left|u_{i\prime }^{m\prime }-(w_{m}^{T}\Phi_{i\prime }+1b_{m})+\xi_{i\prime }^{m\prime }/\mu \right|\right|}_{2}^{2}].$$

We take the derivative with respect to $w_{m}$ and set it equal to zero to solve for $w_{m}$:

(10) $$\begin{array}{cc} & {\left(w_{m}^{*}\right)}^{T}=(\left[\left(t^{m}-1b_{m}+\theta^{m}/\mu \right)\Phi^{T}\right]+\sum_{m\prime =1}^{K}[(u_{\prime }^{m\prime }\\ & -1b_{m}+\xi_{\prime }^{m\prime }/\mu )\Phi_{\prime }^{T}])*{\left(I/\mu +\Phi \Phi^{T}+K\Phi_{\prime }\Phi_{\prime }^{T}\right)}^{-1}, \end{array}$$

where $\Phi =[\Phi_{1},\;\ldots ,\Phi_{N}]\in R^{d_{z}\times N_{t}}$ and $\Phi_{\prime }=[\Phi_{1\prime },\;\ldots ,\Phi_{N\prime }]\in R^{d_{z}\times N_{t}^{\prime }}$. Here $N_{t}=\sum_{i=1}^{N}n_{i}$ and $N_{t}^{\prime }=\sum_{i\prime =1}^{N\prime }n_{i}$ denote the total number of instances which belongs to all classes and *m*th class respectively, and $\Phi_{\prime }$ contains the N′ column blocks of Φ corresponding to the *m*th class.

However, the dimensionality *d_z_* of feature vectors Φ of kernel function can be very large (possibly infinitely large), thus calculating ${\left(I/\mu +\Phi \Phi^{T}+K\Phi_{\prime }\Phi_{\prime }^{T}\right)}^{-1}$ in Equation (10) may not be computationally feasible. In order to derive the scalable solution against arbitrary kernel function, we rewrite Equation (10) into the following matrix form:

(11) $${\left(w_{m}^{*}\right)}^{T}=s_{m}D{\hat{\Phi }}^{T}*{\left(I/\mu +\hat{\Phi }D{\hat{\Phi }}^{T}\right)}^{-1},$$

where $s_{m}=[t^{m}-1b_{m}+\theta^{m}/\mu ,\;1/K\sum_{m\prime =1}^{K}\left(u_{\prime }^{m\prime }-1b_{m}+\xi_{\prime }^{m\prime }/\mu \right)],\;D=[I,\;0;\;0,\;KI]$ and $\hat{\Phi }=[\Phi ,\;\Phi_{\prime }]$. Then we can apply the following kernel trick (Welling, 2013) to Equation (11):

$${\left(P^{-1}+m^{T}R^{-1}m\right)}^{-1}m^{T}R^{-1}=Pm^{T}{\left(mPm^{T}+R\right)}^{-1},$$

which gives:

(12) $${\left(w_{m}^{*}\right)}^{T}=s_{m}{\left({\hat{\Phi }}^{T}\hat{\Phi }+D^{-1}/\mu \right)}^{-1}{\hat{\Phi }}^{T}.$$

In Equation (12), we avoid to compute the feature vectors Φ in the possibly large dimensionality *d_z_*. Instead we need to compute the inner product of feature vectors ${\hat{\Phi }}^{T}\hat{\Phi }\in R^{\left(N_{t}+N_{t}^{\prime })\times (N_{t}+N_{t}^{\prime }\right)}$ which is usually more efficient than directly computing $\Phi \Phi^{T}\in R^{d_{z}\times d_{z}}$.

The algorithm to solve the proposed objective in Equation (4) is summarized in Algorithm 1.

Algorithm 1 The multiblock ADMM updates to optimize Equation (4).

**Data:**  $X\in R^{D\times (n_{1}+\cdots +n_{N})}$ and $Y\in {\left\{-1,1\right\}}^{K\times N}$.

**Hyperparameters:** *C *>* *0, μ>0, ρ>1 and *tolerance* > 0.

**Initialize:** primal variables W,b,E,Q,R,T,U and dual variables Λ,Σ,Θ,Ω,Ξ.

**while** residual >tolerance  **do**

 **for**  m∈K  **do**
 

$$\begin{array}{c} U\mathrm{pdate}\;w_{m}\in W\;by\;Eq.\;(13). \end{array}$$

 

$$\begin{array}{cc} U\mathrm{pdate}\;b_{m}\in b\;by\;b_{m}= & \\ \frac{\sum_{i=1}^{N}\left[t_{i}^{m}-w_{m}^{T}X_{i}+\theta_{i}^{m}/\mu \right]+\sum_{i\prime =1}^{N\prime }\sum_{m=1}^{K}[u_{i\prime }^{m}-w_{m}^{T}X_{i\prime }+\xi_{i\prime }^{m}/\mu ]}{N+KN\prime }. & \end{array}$$

**end for**

**for**  (p,m)∈{N,K}  **do**
 

$$\begin{array}{cc} U\mathrm{pdate}\;e_{p}^{m}\in E\;by\;e_{i}^{m}=\{\begin{array}{cc} n_{i}^{m}-\frac{C}{\mu }y_{i}^{m}\; & \text{when}\;y_{i}^{m}n_{i}^{m}>\frac{C}{\mu },\\ 0 & \text{when}\;0\leq y_{i}^{m}n_{i}^{m}\leq \frac{C}{\mu },\\ n_{i}^{m} & \text{when}\;y_{i}^{m}n_{i}^{m}<0, \end{array} & \\ \text{where}\;n_{i}^{m}=y_{i}^{m}-q_{i}^{m}+r_{i}^{m}-\lambda_{i}^{m}/\mu . & \end{array}$$

 

$$\begin{array}{cc} U\mathrm{pdate}\;q_{p}^{m}\in Q\;by & \\ & q_{i}^{m}=\frac{\left(y_{i}^{m}-e_{i}^{m}+r_{i}^{m}-\lambda_{i}^{m}/\mu +\max(t_{i}^{m})-\sigma_{i}^{m}/\mu \right)}{2}. \end{array}$$

 

$$\begin{array}{cc} U\mathrm{pdate}\;r_{p}^{m}\in R\;by & \\ r_{i}^{m}=\frac{\left(e_{i}^{m}-y_{i}^{m}+q_{i}^{m}+\lambda_{i}^{m}/\mu +\max(u_{i}^{m})-\omega_{i}^{m}/\mu \right)}{2}. & \end{array}$$

** for**  $j\in n_{p}$  **do**
 

$$\begin{array}{cc} & U\mathrm{pdate}\;t_{p,j}^{m}\in T\;by\\ & t_{i,j}^{m}=\{\begin{array}{cc} \frac{\max(\phi_{i}^{m})+q_{i}^{m}+\sigma_{i}^{m}/\mu }{2} & \text{if}\;j=\text{arg max}(\phi_{i}^{m}),\\ \phi_{i,j}^{m}\; & \text{else}, \end{array}\\ & \text{where}\;\phi_{i}^{m}=w_{m}^{T}X_{i}+1b_{m}-\theta_{i}^{m}/\mu . \end{array}$$

 

$$\begin{array}{cc} & U\mathrm{pdate}\;u_{p,j}^{m}\in U\;by\\ & u_{i,j}^{m}=\{\begin{array}{cc} \frac{\max(\psi_{i}^{m})+r_{i}^{m}+\omega_{i}^{m}/\mu }{2} & \text{if}\;j=\text{arg max}(\psi_{i}^{m}),\\ \psi_{i,j}^{m}\; & \text{else}, \end{array}\\ & \text{where}\;\psi_{i}^{m}=w_{y}^{T}X_{i}+1b_{y}-\xi_{i}^{m}/\mu . \end{array}$$

** end for**
 

$$\begin{array}{cc} & U\mathrm{pdate}\;\lambda_{p}^{m},\sigma_{p}^{m},\omega_{p}^{m},\theta_{p}^{m},\xi_{p}^{m}\;by\\ & \lambda_{i}^{m}=\lambda_{i}^{m}+\mu (e_{i}^{m}-(y_{i}^{m}-q_{i}^{m}+r_{i}^{m}));\\ & \sigma_{i}^{m}=\sigma_{i}^{m}+\mu (q_{i}^{m}-\max(t_{i}^{m}));\\ & \omega_{i}^{m}=\omega_{i}^{m}+\mu (r_{i}^{m}-\max(u_{i}^{m}));\\ & \theta_{i}^{m}=\theta_{i}^{m}+\mu (t_{i}^{m}-(w_{m}^{T}X_{i}+1b_{m}));\\ & \xi_{i}^{m}=\xi_{i}^{m}+\mu (u_{i}^{m}-(w_{y}^{T}X_{i}+1b_{y})). \end{array}$$

** end for**

** **Update μ=ρμ.

**end while**

**return**  $(w_{m},\ldots ,w_{K})\in W\;\text{and}\;(b_{1},\ldots ,b_{K})\in b$.

### 3.4 Avoiding calculations of the least-squares problems

As can be seen in Equation (8), the update for $w_{m}$ is reliant on solving a least squares problem in every iteration. However, the least squares solver has complexity $O(Nd^{2})$ and will have to be solved every iteration which may not be computationally feasible if the number of features *d* is very large. To avoid this problem we can instead utilize an optimal line search method (Nie *et al.*, 2014) and update $w_{m}$ via gradient descent:

(13) $$w_{m}=w_{m}-s_{m}\nabla_{w_{m}},$$

where $\nabla_{w_{m}}$ is the analytical gradient of Equation (4) with respect to $w_{m}$:

(14) $$\begin{array}{cc} \nabla_{w_{m}}= & w_{m}-\mu X_{i}\sum_{i=1}^{N}{\left[t_{i}^{m}-w_{m}^{T}X_{i}-1b_{m}+\theta_{i}^{m}/\mu \right]}^{T}\\ & -\mu X_{i^{\prime }}\sum_{i^{\prime }=1}^{N^{\prime }}\sum_{m^{\prime }=1}^{K}{\left[u_{i^{\prime }}^{m^{\prime }}-w_{m}^{T}X_{i^{\prime }}-1b_{m}+\xi_{i^{\prime }}^{m^{\prime }}/\mu \right]}^{T}, \end{array}$$

and it can be used to define a minimization:

(15) $$\begin{array}{cc} & s_{m}^{*}=\underset{s_{m}}{\text{arg min}}\;\frac{1}{2}{\left|\left|w_{m}^{T}-s_{m}\nabla_{w_{m}}^{T}\right|\right|}_{2}^{2}\\ & +\frac{\mu }{2}\sum_{i=1}^{N}[{\left|\left|t_{i}^{m}-(w_{m}^{T}-s_{m}\nabla_{w_{m}}^{T})X_{i}-1b_{m}+\theta_{i}^{m}/\mu \right|\right|}_{2}^{2}]\\ & +\sum_{i\prime =1}^{N\prime }\sum_{m=1}^{K}[\frac{\mu }{2}{\left|\left|u_{i\prime }^{m}-(w_{m}^{T}-s_{m}\nabla_{w_{m}}^{T})X_{i\prime }-1b_{m}+\xi_{i\prime }^{m}/\mu \right|\right|}_{2}^{2}], \end{array}$$

in terms of *s_m_* instead of $w_{m}$. Differentiating Equation (15) with respect to *s_m_*, setting the result equal to zero gives:

(16) $$s_{m}^{*}=\frac{\left(w_{m}^{T}-\mu \sum_{i=1}^{N}{\hat{t}}_{i}^{m}X_{i}^{T}-\mu \sum_{i\prime =1}^{N\prime }\sum_{m\prime }^{K}{\hat{u}}_{i\prime }^{m\prime }X_{i\prime }^{T}\right)\nabla_{w_{m}}}{\nabla_{w_{m}}^{T}\left(I+\mu \sum_{i=1}^{N}X_{i}X_{i}^{T}+\mu K\sum_{i\prime =1}^{N\prime }X_{i\prime }X_{i\prime }^{T}\right)\nabla_{w_{m}}}$$

where ${\hat{t}}_{i}^{m}=t_{i}^{m}-w_{m}^{T}X_{i}-1b_{m}+\theta_{i}^{m}/\mu$ and ${\hat{u}}_{i\prime }^{m\prime }=u_{i\prime }^{m\prime }-w_{m}^{T}X_{i\prime }-1b_{m}+\xi_{i\prime }^{m\prime }/\mu$. Finally we plug Equations (14) and (16) into Equation (13) to earn an efficient update equation which avoids the least squares problem in Equation (8). The time complexity of the proposed method is $O(Nd\bar{n})$, where $\bar{n}$ is the average number of instances per bag. The number of instances $\bar{n}$ is typically smaller than the number of features *d* (the multiple of 162 in our experiments), therefore our model with the solution in Equation (13) (inexact *pdMISVM*) is more scalable compared to Equation (8) (exact *pdMISVM*).

## 4 Results

In our experiments, we evaluate the classification performance and scalability of the proposed exact and inexact *pdMISVM* implementations. The scalability of *pdMISVM* is assessed across the increasing number of bags and features. Regarding the interpretability of our model, we also identify the disease relevant patches (instances) of each bag (image).

### 4.1 Benchmarks and hyperparameters

The classification performance and scalability of *pdMISVM* is compared against the following standard MIL benchmarks:

- A SIL method that assigns the bags’ labels to all instances during training and produces the maximum response for each bag/class pair at testing time for the training bag’s instances.
- The two bag-based methods; Normalized Set Kernel (NSK) and Statistics Kernel (STK) (Gärtner *et al.*, 2002), which map the entire bag to a single-instance by a way of kernel function.
- An iterated discrimination Axis-Parallel Rectangles algorithm (APR) (Dietterich *et al.*, 1997): the APR is a MIL model which starts from a single positive instance and grows the APR by expanding it to cover the remaining positive instances.
- The two multi-instance deep learning methods: The mi-Net and MI-Net (Wang *et al.*, 2018) approach to the MIL problem in a way of instance space and embedded space (learning vectorial representation of the bag) paradigm respectively.
- The two attention mechanism-based MIL models: Ilse *et al.* (2018) (AMIL) calculate the parameterized attention (importance) score for each instance to generate the probability distribution of bag labels. Shi *et al.* (2020) (LAMIL) propose to learn the instance scores and predictions jointly by integrating the attention mechanism with the loss function.

For these SVM models, the regularization tradeoff is set to 1.0. For the exact and inexact *pdMISVM*, the regularization tradeoff *C* is set to 1e−3 and 1e+4 respectively, the tolerance is set to 1e−5 for both, and *μ* is initialized with 1e−10 and 1e−8 respectively. We use the radial basis kernel function for all SVM models (except inexact *pdMISVM* which uses linear kernel). For the deep learning models (mi-Net, MI-Net, AMIL and LAMIL), we use the same hyperparameters as in their articles.

### 4.2 Classification performance

In this section, we evaluate the classification models to investigate whether our exact/inexact *pdMISVM* achieves the better or comparable performance to the best performing classical or recent models. In Table 1, we report the performance of our *pdMISVM* compared against the other MIL algorithms in the classification of benign/malignant bags. For each model, we provide the precision, recall, F1-score, accuracy and balanced accuracy (BACC) across the 10 6-fold cross-validation experiments (six repetitions per experiment).

**Table 1.** The classification performance of our *pdMISVM* and competing models with the different magnification levels

Model	Magnification	Precision	Recall	F1Score	Accuracy	BACC
SIL	**40×**	0.874 ± 0.018	0.752 ± 0.043	0.829 ± 0.030	0.787 ± 0.036	0.808 ± 0.032
NSK	**40×**	0.906 ± 0.016	0.902 ± 0.025	0.904 ± 0.014	0.868 ± 0.019	0.848 ± 0.022
STK	**40×**	**0.911 ± 0.031**	0.907 ± 0.036	0.908 ± 0.017	0.875 ± 0.021	0.857 ± 0.024
APR	**40×**	0.881 ± 0.019	0.813 ± 0.034	0.856 ± 0.036	0.793 ± 0.024	0.817 ± 0.035
mi-Net	**40×**	0.883 ± 0.016	0.872 ± 0.037	0.883 ± 0.025	0.836 ± 0.032	0.850 ± 0.027
Mi-Net	**40×**	0.891 ± 0.070	0.884 ± 0.045	0.887 ± 0.023	0.852 ± 0.029	0.842 ± 0.030
AMIL	**40×**	0.901 ± 0.025	0.897 ± 0.019	0.899 ± 0.031	0.870 ± 0.031	0.861 ± 0.024
LAMIL	**40×**	0.896 ± 0.041	0.900 ± 0.023	0.894 ± 0.047	0.863 ± 0.024	0.859 ± 0.027
Ours	**40×**	0.894 ± 0.018	**0.924 ± 0.036**	0.903 ± 0.023	0.853 ± 0.028	0.842 ± 0.034
Ours (inexact)	**40×**	0.902 ± 0.014	0.916 ± 0.024	**0.916 ± 0.009**	**0.879 ± 0.009**	**0.863 ± 0.023**
SIL	**100×**	0.908 ± 0.014	0.797 ± 0.034	0.848 ± 0.023	0.804 ± 0.027	0.808 ± 0.025
NSK	**100×**	0.918 ± 0.019	0.926 ± 0.008	0.922 ± 0.011	**0.892 ± 0.013**	0.872 ± 0.017
STK	**100×**	0.895 ± 0.024	0.929 ± 0.023	0.911 ± 0.010	0.876 ± 0.012	0.844 ± 0.012
APR	**100×**	0.896 ± 0.025	0.854 ± 0.039	0.879 ± 0.032	0.818 ± 0.031	0.812 ± 0.034
mi-Net	**100×**	0.860 ± 0.019	0.917 ± 0.025	0.889 ± 0.019	0.879 ± 0.032	0.862 ± 0.023
Mi-Net	**100×**	0.876 ± 0.026	0.928 ± 0.029	0.891 ± 0.025	0.869 ± 0.027	0.870 ± 0.019
AMIL	**100×**	0.889 ± 0.027	0.935 ± 0.038	0.914 ± 0.039	0.867 ± 0.029	0.869 ± 0.029
LAMIL	**100×**	0.898 ± 0.046	0.924 ± 0.035	0.910 ± 0.041	0.870 ± 0.034	0.859 ± 0.032
Ours	**100×**	0.873 ± 0.016	**0.944 ± 0.019**	0.919 ± 0.009	0.883 ± 0.017	0.864 ± 0.026
Ours (inexact)	**100×**	**0.923 ± 0.025**	0.942 ± 0.022	**0.925 ± 0.020**	0.891 ± 0.024	**0.876 ± 0.041**
SIL	**200×**	0.903 ± 0.015	0.812 ± 0.018	0.863 ± 0.007	0.821 ± 0.013	0.826 ± 0.016
NSK	**200×**	0.902 ± 0.020	0.935 ± 0.022	0.923 ± 0.016	0.893 ± 0.022	0.867 ± 0.025
STK	**200×**	0.898 ± 0.025	0.927 ± 0.020	0.922 ± 0.011	0.892 ± 0.017	0.871 ± 0.025
APR	**200×**	0.889 ± 0.021	0.897 ± 0.024	0.895 ± 0.013	0.857 ± 0.018	0.864 ± 0.027
mi-Net	**200×**	0.879 ± 0.021	0.909 ± 0.032	0.891 ± 0.028	0.876 ± 0.021	0.846 ± 0.023
Mi-Net	**200×**	0.885 ± 0.020	0.918 ± 0.029	0.896 ± 0.025	0.885 ± 0.019	0.851 ± 0.024
AMIL	**200×**	**0.905 ± 0.024**	0.918 ± 0.031	0.900 ± 0.027	0.881 ± 0.024	0.849 ± 0.021
LAMIL	**200×**	0.891 ± 0.031	0.914 ± 0.037	0.907 ± 0.030	0.875 ± 0.024	0.853 ± 0.028
Ours	**200×**	0.903 ± 0.017	**0.936 ± 0.023**	**0.924 ± 0.012**	**0.898 ± 0.017**	**0.872 ± 0.026**
Ours (inexact)	**200×**	0.890 ± 0.019	0.931 ± 0.017	0.918 ± 0.012	0.889 ± 0.021	0.859 ± 0.020
SIL	**400×**	0.860 ± 0.021	0.744 ± 0.042	0.819 ± 0.027	0.778 ± 0.032	0.797 ± 0.029
NSK	**400×**	0.890 ± 0.022	0.910 ± 0.013	0.886 ± 0.009	0.863 ± 0.014	0.836 ± 0.025
STK	**400×**	0.889 ± 0.024	0.898 ± 0.029	0.893 ± 0.014	0.854 ± 0.020	0.832 ± 0.023
APR	**400×**	0.891 ± 0.025	0.803 ± 0.038	0.871 ± 0.033	0.816 ± 0.028	0.819 ± 0.036
mi-Net	**400×**	0.837 ± 0.021	0.901 ± 0.025	0.864 ± 0.029	0.831 ± 0.031	0.822 ± 0.024
Mi-Net	**400×**	0.849 ± 0.020	0.895 ± 0.026	0.871 ± 0.024	0.841 ± 0.028	0.820 ± 0.025
AMIL	**400×**	0.852 ± 0.019	0.897 ± 0.022	0.880 ± 0.023	0.846 ± 0.024	0.818 ± 0.027
LAMIL	**400×**	0.867 ± 0.025	0.889 ± 0.029	0.891 ± 0.031	0.857 ± 0.023	0.819 ± 0.029
Ours	**400×**	**0.909 ± 0.012**	**0.932 ± 0.016**	**0.899 ± 0.012**	**0.868 ± 0.016**	**0.838 ± 0.018**
Ours (inexact)	**400×**	0.875 ± 0.023	0.923 ± 0.019	0.898 ± 0.015	0.858 ± 0.019	0.823 ± 0.020

*Note*: The reported metrics and their standard deviations are calculated across 10 6-fold cross-validation experiments. The best scores are highlighted in bold font.

From the results reported in Table 1, the proposed exact/inexact *pdMISVM* show promising performance across the various magnification levels. In particular, our exact *pdMISVM* outperforms the other models based on recall. A high recall rate is critical in the medical domain, as false negatives may result the serious consequences. This result shows the clinical utility of our model as it is crucial not to miss a malignant tumor in the diagnosis. When the SIL model is compared to the other MIL models, SIL performed the worst because it is difficult to accurately classify labels from individual patches. For example, evidence of malignancy may appear only in some patches of the bag. In this case, it is difficult to classify a patch as a malignancy from a patch where no evidence of a malignant tumor appeared. Our experimental results support the assumption that MIL models will classify better than SIL model.

Interestingly, our exact *pdMISVM* performs better than the inexact version at the smaller magnification levels, and while the opposite results are observed in the larger magnification levels. These results show that the classification pattern of *pdMISVM* can vary depending on the choice of optimization approach, just like the impact of the optimization algorithm on the deep learning models (Wang *et al.*, 2019). Although our derivation for inexact *pdMISVM* does not obtain the exact optimal solution of the MISVM objective in Equation (1), our experimental results show that the inexact solution may improve the classification performance when compared to the exact solution. This is well supported by the previous finding (Chang *et al.*, 2008) that some implementations of SVM achieve the highest accuracy before the objective reaches its minimum. Our exact/inexact *pdMISVM* has gained the overall improved accuracy/BACC as well, and this validates their usefulness in the field of MIL and the early detection of a malignant tumor.

### 4.3 The scalability against bags and features

**Fig. 3.**
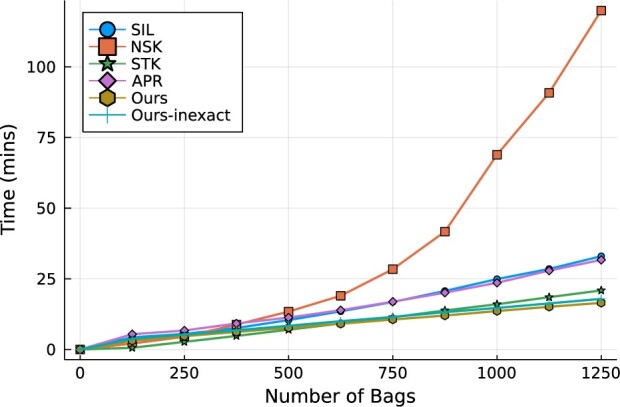
Computation time over the increasing number of bags. The number of features is fixed at 162

**Fig. 4.**
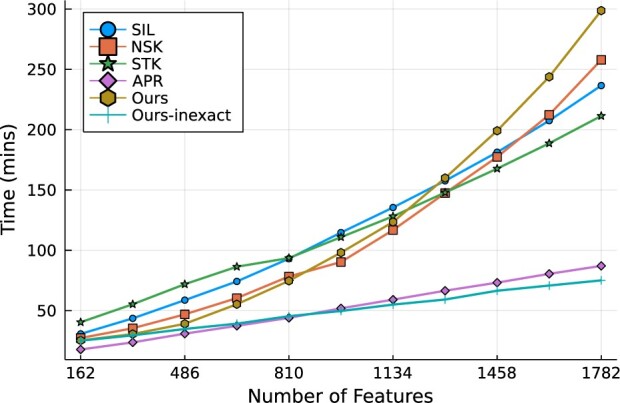
Computation time over the increasing number of features. The number of features are controlled by concatenating the multiple patches of image

The main contribution of this study is that the derived Algorithm 1 scales to the large dataset. In this timing experiment, our goal is to verify the analytical complexity calculated in Section 3.4 on the real world dataset. We plot the training time of the classifiers on the BreaKHis dataset to verify this improved scalability against the number of bags in Figure 3 and the number of features in Figure 4. In this timing experiment, we use the linear kernel function for all SVM models. The deep learning models are excluded in this experiment as their training times exceed the reasonable limit (5 h). In Figure 3, the running time of NSK increases rapidly while the other models maintain the linear trend. Our *pdMISVM* outperforms the other models in training a large number of bags. This result validates the superior scalability of the proposed primal-dual approach over the other SVM models which rely on repeatedly solving a quadratic programming problem.

Despite the fact that the initial derivation with Equation (8) scales well with respect to the bags, the update for $w_{k}$ in Equation (8) requires solving a least-squares problem that scales quadratically as the number of features *d* increases. To tackle this difficulty, we adapt an optimal line search method in Equation (13) to achieve the linear complexity against the number of features. In Figure 4, we compare the training time of the exact/inexact versions of our models to the other competing models. Among all models, Ours-inexact and APR spend the smallest training time when trained with the large number of features. Interestingly, inexact variation of *pdMISVM* scales significantly better than the exact *pdMISVM* against the increasing number of features where the number of bags is fixed at 1000. This is well represented by the analytical complexity of the two derivations ($O(Nd^{2})$ versus $O(Nd\bar{n})$) as discussed in Section 3.4.

### 4.4 Patch identification

**Fig. 5.**
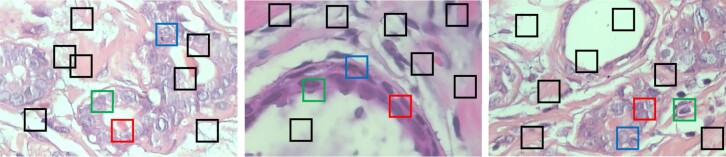
The identified patches in the *adenosis (benign)* images, where the red, green and blue boxes denote the first, second and third most important patches

**Fig. 6.**
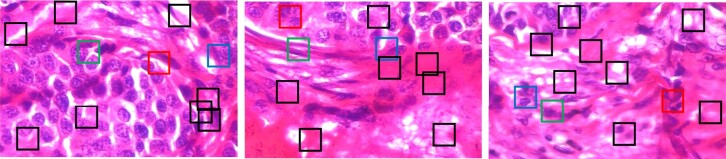
The identified patches in the *ductal carcinoma (malignant)* images, where the red, green and blue boxes denote the first, second and third most important patches

Along with the improved prediction performance and scalability, our model *pdMISVM* can identify disease-relevant locations. The interpretability is crucial as it can add confidence to the generated predictions and help clinicians use histopathological references to make a diagnosis. We calculate the patch-wise importance $\max(W^{T}x_{i}^{j}+b)$ which is the response of *j*th patch to the decision function in Equation (2). Figures 5 and 6 show the identified patches in the benign and malignant images at 400× magnification level. In Figures 5 and 6, the 10 boxes (patches) represent the 10 instances of each bag (image).

The patches identified by our model are in accordance with the clinical insights. The color, shape and size morphologic abnormalities of the nuclei are regarded as the key characteristics that categorize a digitized biopsy as cancerous or non-cancerous (Rajbongshi *et al.*, 2018). For example, in the third image in Figure 5, our model highlights the regions containing the cell’s nuclei. From the identified patches, our model can reveal that the nuclear to cell volume ratio is consistent throughout which is a distinctive feature of non-carcinoma (Jevtić and Levy, 2014). Because of this, our model correctly classifies the bag as benign. A previous study (Kumar *et al.*, 2015) explains that a disorganized arrangement of cells is one of the characteristics of cancerous cells. In the second image in Figure 5, our model identifies a continuous, organized distribution of cells so this is another indication that our model was correct in labeling this image as benign. For the three malignant samples in Figure 6, our model focuses on the variation in the size and shape of nuclei. Based on the literature (Fischer, 2020), the loss of normal morphology and large/varying shape of nuclei are essential for the diagnosis of malignancy in the practice of surgical pathology. The accurately identified regions validate the correctness of our model in the histopathological image classification and add value to its clinical practicability.

## 5 Discussion

We demonstrated that the MIL SVM can detect the malignancy in the patches. With the development of image acquisition technology, and it has become crucial to train the models with the large amount of images to improve classification performance. Accordingly, scalability has emerged as a major issue, and the improved scalability can increase the performance and decrease the cost in response to the system processing demands of CAD. Therefore, this study proposes a new optimization method for SVM with improved scalability. The proposed method reduces the computational complexity against the large number of features of instances by approximating the optimal point of SVM, but nevertheless, the experimental results show that the classification performance of SVM is not sacrificed, and rather improved in certain cases (in the lower magnification levels). In addition, the permutation invariant property is satisfied in Equation (1), which is desirable in the MIL. The proposed optimization method can be applied regardless of whether the kernel function is used, however we plan to deal with the improved scalability of kernelized SVM in the future study. In this study, we have sampled the patches of WSIs at the random locations, and we plan to integrate the attention mechanism to automatically sample the patches important for malignant tumor detection. In this study, we propose a general framework for MIL and the other models stemming from our approach can be flexibly applied to solve the various MIL problems.

## 6 Conclusion

The improvement of the scalability of methods is attracting more attention from machine learning studies as the amount of available data is increasing due to the development of data mining technologies. In this work, we present a novel *Primal-Dual Multi-Instance SVM* method and the associated derivations, which scale to a large number of bags and features. We have conducted extensive experiments on the BreaKHis dataset to show the promising performance and scalability of the proposed method when compared to the traditional SVM-based MIL techniques. In addition to the improved classification performance and scalability, the key patches for the classification identified by our model are well supported by previous medical studies. The experimental results illustrate the clinical utility of our approach on the detection of cancerous abnormalities in a large dataset to prevent the progression of breast cancer in a patient.

## Funding

This work was supported in part by the National Science Foundation (NSF) under the grants of Information and Intelligent Systems (IIS) [1652943, 1849359], Computer and Network Systems (CNS) [1932482].

*Conflict of Interest*: none declared.

## Supplementary Material

btac267_Supplementary_Data

## References

[btac267-B1] Andrews S. et al (2002) Support vector machines for multiple-instance learning. In: Advances in neural information processing systems (NIPS), Vol. 2. Citeseer, pp. 561–568.

[btac267-B2] Brand L. et al (2021a) A linear primal-dual multi-instance svm for big data classifications. In: *2021 IEEE International Conference on Data Mining (ICDM)*. IEEE, pp. 21–30.

[btac267-B3] Brand L. et al (2021b) A multi-instance support vector machine with incomplete data for clinical outcome prediction of covid-19. In: *Proceedings of the 12th ACM Conference on Bioinformatics, Computational Biology, and Health Informatics*, pp. 1–6.

[btac267-B4] Bunescu R.C. , Mooney R.J. (2007) Multiple instance learning for sparse positive bags. In *Proceedings of the 24th international conference on Machine learning*, pp. 105–112.

[btac267-B5] CDC. (2020) *The Basics on Hereditary Breast and Ovarian Cancer—CDC*. https://www.cdc.gov/genomics/disease/breast_ovarian_cancer/basics_hboc.htm (1 August 2021, date last accessed).

[btac267-B6] Chang K.-W. et al (2008) Coordinate descent method for large-scale l2-loss linear support vector machines. J. Mach. Learn. Res., 9, 1369–1398.

[btac267-B7] Dietterich T.G. et al (1997) Solving the multiple instance problem with axis-parallel rectangles. Artif. Intell., 89, 31–71.

[btac267-B8] Fischer E.G. (2020) Nuclear morphology and the biology of cancer cells. Acta Cytol., 64, 511–519. 32570234 10.1159/000508780

[btac267-B9] Gärtner T. et al (2002) Multi-instance kernels. In: *International Conference on Machine Learning (ICML)*, Vol. 2, p. 7.

[btac267-B10] Guo Z. et al (2010) A completed modeling of local binary pattern operator for texture classification. IEEE Trans. Image Process., 19, 1657–1663. 20215079 10.1109/TIP.2010.2044957

[btac267-B11] Gurcan M.N. et al (2009) Histopathological image analysis: a review. IEEE Rev. Biomed. Eng., 2, 147–171. 20671804 10.1109/RBME.2009.2034865 PMC2910932

[btac267-B12] Hamilton N.A. et al (2007) Fast automated cell phenotype image classification. BMC Bioinformatics, 8, 110–118. 17394669 10.1186/1471-2105-8-110 PMC1847687

[btac267-B13] Haralick R.M. (1979) Statistical and structural approaches to texture. Proc. IEEE, 67, 786–804.

[btac267-B14] Haralick R.M. et al (1973) Textural features for image classification. IEEE Trans. Syst, Man, Cybern., SMC-3, 610–621.

[btac267-B15] Hong M. , Luo Z.-Q. (2017) On the linear convergence of the alternating direction method of multipliers. Math. Program., 162, 165–199.

[btac267-B16] Ilse M. et al (2018) Attention-based deep multiple instance learning. In: *International Conference on Machine Learning*. PMLR, pp. 2127–2136.

[btac267-B17] Jevtić P. , Levy D.L. (2014) Mechanisms of nuclear size regulation in model systems and cancer. Cancer Biol. Nuclear Envelope, 773, 537–569. 10.1007/978-1-4899-8032-8_25 24563365

[btac267-B18] Kahya M.A. et al (2017) Classification of breast cancer histopathology images based on adaptive sparse support vector machine. J. Appl. Math. Bioinf., 7, 49.

[btac267-B19] Khotanzad A. , Hong Y.H. (1990) Invariant image recognition by Zernike moments. IEEE Trans. Pattern Anal. Machine Intell., 12, 489–497.

[btac267-B20] Krizhevsky A. et al (2012) Imagenet classification with deep convolutional neural networks. Adv. Neural Inf. Process. Syst., 25, 1097–1105.

[btac267-B21] Kumar M. , Rath S.K. (2015) Classification of microarray using mapreduce based proximal support vector machine classifier. Knowledge Based Syst., 89, 584–602.

[btac267-B22] Kumar R. et al (2015) Detection and classification of cancer from microscopic biopsy images using clinically significant and biologically interpretable features. J. Med. Eng., 2015, 457906. 27006938 10.1155/2015/457906 PMC4782618

[btac267-B23] Nie F. et al (2014) New primal SVM solver with linear computational cost for big data classifications. In: *Proceedings of the 31st International Conference on International Conference on Machine Learning*, Vol. 32, pp. II–505.

[btac267-B24] Ojala T. et al (2002) Multiresolution gray-scale and rotation invariant texture classification with local binary patterns. IEEE Trans. Pattern Anal. Machine Intell., 24, 971–987.

[btac267-B25] Ojansivu V. , Heikkilä J. (2008) Blur insensitive texture classification using local phase quantization. In: International Conference on Image and Signal Processing. Springer, pp. 236–243.

[btac267-B26] Otsu N. (1979) A threshold selection method from gray-level histograms. IEEE Trans. Syst, Man Cybern., 9, 62–66.

[btac267-B27] Peng X. et al (2016) L1-norm loss based twin support vector machine for data recognition. Inf. Sci., 340-341, 86–103.

[btac267-B28] Rajbongshi N. et al (2018) Analysis of morphological features of benign and malignant breast cell extracted from FNAC microscopic image using the Pearsonian system of curves. J. Cytol., 35, 99–104. 29643657 10.4103/JOC.JOC_198_16 PMC5885612

[btac267-B29] Shawe-Taylor J. et al (2004) Kernel Methods for Pattern Analysis. Cambridge University Press, New York, NY.

[btac267-B30] Shi X. et al (2020) Loss-based attention for deep multiple instance learning. In: *Proceedings of the AAAI Conference on Artificial Intelligence*, Vol. 34, pp. 5742–5749.

[btac267-B31] Spanhol F.A. et al (2015) A dataset for breast cancer histopathological image classification. IEEE Trans. Biomed. Eng., 63, 1455–1462. 26540668 10.1109/TBME.2015.2496264

[btac267-B32] Spanhol F.A. et al (2016) Breast cancer histopathological image classification using convolutional neural networks. In: *2016 International Joint Conference on Neural Networks (IJCNN)*. IEEE, pp. 2560–2567.

[btac267-B33] Sudharshan P. et al (2019) Multiple instance learning for histopathological breast cancer image classification. Expert Syst. Appl., 117, 103–111.

[btac267-B34] Titoriya A. , Sachdeva S. (2019) Breast cancer histopathology image classification using Alexnet. In: *2019 4th International Conference on Information Systems and Computer Networks (ISCON)*. IEEE, pp. 708–712.

[btac267-B35] van der Laak J. et al (2021) Deep learning in histopathology: the path to the clinic. Nat. Med., 27, 775–784. 33990804 10.1038/s41591-021-01343-4

[btac267-B36] Wang J. , Zhao L. (2021) Nonconvex generalization of Alternating Direction Method of Multipliers for nonlinear equality constrained problems. Results in Con. Optim., 2, 100009. 10.1016/j.rico.2021.100009.

[btac267-B37] Wang H. et al (2011) Learning instance specific distance for multi-instance classification. In: *Twenty-Fifth AAAI Conference on Artificial Intelligence, San Francisco, CA, USA*. Association for the Advancement of Artificial Intelligence (AAAI), Palo Alto, California, USA. pp. 507–512.

[btac267-B38] Wang X. et al (2018) Revisiting multiple instance neural networks. Pattern Recognit., 74, 15–24.

[btac267-B39] Wang Y. et al (2019) Assessing optimizer impact on DNN model sensitivity to adversarial examples. IEEE Access, 7, 152766–152776.

[btac267-B40] Wei X.-S. et al (2014) Scalable multi-instance learning. In: *2014 IEEE International Conference on Data Mining*. IEEE, pp. 1037–1042.

[btac267-B41] Welling M. (2013) Kernel ridge regression. Max Welling’s Classnotes Mach. Learn., 1–3.

[btac267-B42] Zheng B. et al (2014) Breast cancer diagnosis based on feature extraction using a hybrid of k-means and support vector machine algorithms. Exp. Syst. Appl., 41, 1476–1482.

