# Supplementary material for: Scaling multi-instance support vector machine to breast cancer detection on the BreaKHis dataset
Source: Bioinformatics. 2022 Jun 27;38(Suppl 1):i92–i100. doi: 10.1093/bioinformatics/btac267 (PMC9235475; doi:10.1093/bioinformatics/btac267)
Supplement: btac267_Supplementary_Data [file btac267_supplementary_data.pdf]

Appendix: Scaling Multi-Instance Support Vector Machine to Breast Cancer Detection on the BreaKHis Dataset

S1 Derivation Details

In this section, we provide the derivation of solution for each variable discussed in Section 3.3. For each variable $\mathbf{X}$, we formulize the variable specific objective by keeping the terms containing $\mathbf{X}$ in Eq. (4). Then we take the derivative of the objective respect to each $x_i^j \in \mathbf{X}$ and set it to zero matrix to earn the update for $\mathbf{X}$.

E update. Dropping terms from Eq. (4), that do not contain $\mathbf{E}$ and decoupling element-wise gives $K \times N$ problems

$$e_i^m = \underset{e_i^m}{\arg\min}\, C\left(y_i^m e_i^m\right)_+ + \frac{\mu}{2}\left(e_i^m - n_i^m\right)^2 \ , \tag{S1}$$

where $n_i^m = y_i^m - q_i^m + r_i^m - \frac{\lambda_i^m}{\mu}$. Equation (S1) can be differentiated with respect to e_i^m via the sub-gradient method, and solved in three cases

$$e_i^m = \begin{cases} n_i^m - \frac{C}{\mu} y_i^m & \text{when} \ \ y_i^m n_i^m > \frac{C}{\mu} \ , \\ 0 & \text{when} \ \ 0 \leq y_i^m n_i^m \leq \frac{C}{\mu} \ , \\ n_i^m & \text{when} \ \ y_i^m n_i^m < 0 \ , \end{cases} \ . \tag{S2}$$

Q & R update. Keeping only terms with $\mathbf{Q}$ in Eq. (4) and decoupling element-wise gives $K \times N$ problems

$$q_i^m = \underset{q_i^m}{\arg\min}\ \left(e_i^m - y_i^m + q_i^m - r_i^m + \lambda_i^m/\mu\right)^2 + \left(q_i^m - \max\left(\mathbf{t}_i^m\right) + \sigma_i^m/\mu\right)^2 \ . \tag{S3}$$

Taking the derivative of Eq. (S3) with respect to q_i^m, setting the result equal to zero, and solving for q_i^m gives the update

$$q_i^m = \frac{\left(y_i^m - e_i^m + r_i^m - \lambda_i^m/\mu + \max\left(\mathbf{t}_i^m\right) - \sigma_i^m/\mu\right)}{2} \ . \tag{S4}$$

Following the same steps for each $r_i^m \in \mathbf{R}$ we derive the element-wise updates

$$r_i^m = \frac{\left(e_i^m - y_i^m + q_i^m + \lambda_i^m/\mu + \max\left(\mathbf{u}_i^m\right) - \omega_i^m/\mu\right)}{2} \ . \tag{S5}$$

T & U update. Keeping terms in Eq. (4) containing $\mathbf{T}$ and decoupling across K and N gives the following

$$\mathbf{t}_i^m = \underset{\mathbf{t}_i^m}{\arg\min}\ \left(q_i^m - \max\left(\mathbf{t}_i^m\right) + \sigma_i^m/\mu\right)^2 + \left\|\mathbf{t}_i^m - \left(\mathbf{w}_m^T \mathbf{X}_i + \mathbf{1} b_m\right) + \theta_i^m/\mu\right\|_2^2 \ , \tag{S6}$$

which can be further decoupled element-wise for each $t_{i,j}^m \in \mathbf{t}_i^m$ giving $K \times N \times (n_1 + \cdots + n_N)$ problems

$$t_{i,j}^m = \underset{t_{i,j}^m}{\arg\min} \begin{cases} \left(q_i^m - t_{i,j}^m + \sigma_i^m/\mu\right)^2 + \left(t_{i,j}^m - \phi_{i,j}^m\right)^2 \\ \text{when } t_{i,j}^m = \max\left(\mathbf{t}_i^m\right) , \\ \left(t_{i,j}^m - \phi_{i,j}^m\right)^2 \ \text{else} \ , \end{cases} \tag{S7}$$

where $\boldsymbol{\phi}_i^m = \mathbf{w}_m^T \mathbf{X}_i + \mathbf{1} b_m - \boldsymbol{\theta}_i^m/\mu$. Taking the derivative of Eq. (S7) with respect to $t_{i,j}^m$, setting the result equal to zero, and solving for $t_{i,j}^m$, gives the updates

$$t_{i,j}^m = \begin{cases} \frac{\max(\boldsymbol{\phi}_i^m) + q_i^m + \sigma_i^m/\mu}{2} & \text{if } j = \arg\max(\boldsymbol{\phi}_i^m) \ , \\ \phi_{i,j}^m & \text{else} \ , \end{cases} \ . \tag{S8}$$

This same strategy is applied to derive the element-wise updates of $\mathbf{U}$, giving

$$u_{i,j}^m = \begin{cases} \frac{\max(\boldsymbol{\psi}_i^m) + r_i^m + \omega_i^m/\mu}{2} & \text{if } j = \arg\max(\boldsymbol{\psi}_i^m) \ , \\ \psi_{i,j}^m & \text{else} \ , \end{cases} \ . \tag{S9}$$

where $\boldsymbol{\psi}_i^m = \mathbf{w}_y^T \mathbf{X}_i + \mathbf{1} b_y - \boldsymbol{\xi}_i^m/\mu$. The associated dual variable updates are provided in Algorithm 1.
